# Danger of Subcutaneous Injections

**Published:** 1865-12

**Authors:** 


### DANGER OF SUBCUTANEOUS INJECTIONS.

Prof. Nassbaum, of Munich, has just published an interesting account of an accident which happened to himself. Suffering from neuralgia, he had injected morphia under his own skin more that 2000 times — sometimes to the extent of five grains of morphia in twenty-four hours. Two months ago he injected two grains acetate of morphia dissolved in fifteen minims of water, and accidentally sent it direct into a subcutaneous vein instead of into the cellular tissue. He gives a graphic account of his dangerous position for two hours, after which the effect passed off. He has seen similar effects in a small degree in two of his patients, and the practical lessons are, that as it may be impossible to avoid veins at all times, and one may be punctured unawares, subcutaneous injection should always be done *very slowly*. The effects are so instantaneous that the syringe can be stopped at the first sign of danger, and some of the injected fluid, mixed with blood, may even be sucked out again by the syringe. It is very remarkable how the effects of the same dose of the same substance differ when injected directly into a vein and mixed with venous blood, and when they filter into the blood from the cellular tissue through the unbroken coats of the vessels. —*Medical Times & Gazette.*

